# Supplementary material for: Automated Detection of Candidate Subjects With Cerebral Microbleeds Using Machine Learning
Source: Front Neuroinform. 2022 Jan 20;15:777828. doi: 10.3389/fninf.2021.777828 (PMC8811357; doi:10.3389/fninf.2021.777828)
Supplement: Supplementary file 1 [file Data_Sheet_1.pdf]

Supplementary material - Automated Detection of Candidate Subjects with Cerebral Microbleeds using Machine Learning

1 Cerebral microbleed (CMB) mimics and their characteristics

CMB mimics	Description and characteristics
Cavernous malformations	Visible on T2-weighted images (subtypes I - IV). Leakage of blood from the vessels at various stages of degradation [1], with subtype II lesions appearing with popcorn-like structure (looks similar to ringing artefacts found on T2*-weighted images). Subtype - IV lesions are punctate and are more difficult to differentiate from CMBs [2].
Haemorrhagic micrometastases	Metastatic melanoma often can bleed and appear hypointense on T2*-weighted images since melanin is paramagnetic, but has concomitant hyperintensities on T1-weighted images surrounded by oedema. Note that other than metastatic melanoma, other haemorrhagic metastases are possible. However, small non-oedemous lesions are difficult to distinguish from CMBs.
Diffuse axonal injury	Concomitant presence of abnormalities (e.g. skull fracture and contusions) [2, 3]. The clinical history of subjects is recommended for distinguishing from CMBs.
Small haemorrhages near infarction and ICH areas	Visible on T2-weighted, T2*-weighted, FLAIR, DWI sequences. Sometimes, they differ from CMBs in size. Would be easier to use infarctions as exclusion biomarkers.
Flow voids	Do not show blooming effect. They have linear/ curvilinear tubular structures extending through contiguous slices, usually appear in the cortical regions and are visible on T2-weighted images [2].
Calcifications	They are usually found in the basal ganglia. However, calcifications could also occur in choroid plexuses and the pineal gland. They appear as hyperintense blobs on CT images [4].
Partial volume artefacts (air-bone interfaces)	Do not show blooming effects. Particularly seen in frontal and temporal lobes (orbit and mastoid bones) and at the edges of the cerebellum. Distinguishable from CMBs using location priors and observation of contiguous slices.

Table S1: CMB mimics and their distinguishing characteristics [1, 2].

2 Performance metrics for comparison of imaging and non-imaging based methods on the UKBB dataset

Table S2 shows the performance metrics for comparison of imaging and non-imaging based methods on the UKBB dataset along with the threshold values.

Table S2: Best performance values determined from the ROC curves for methods based on imaging (I) and non-imaging (NI) data, using the UKBB as the evaluation dataset (section 4.2). Best results in each category highlighted in bold. Sub. TPR: subject-level true positive rate (TPR), Sub. Spec: subject-level specificity, Sub. Acc: subject-level accuracy. For the final category (NI+I), the CMB lesion counts were determined by the proposed pipeline.

**Methods**	**Sub. TPR**	**Sub. Spec**	**Sub. Acc**	**Threshold**
**Demographic/clinical factors considered individually**				
Age	0.34	0.55	0.44	62.7 yrs
**Diastolic BP**	0.37	**0.72**	**0.54**	93.2 mmHg
Systolic BP	**0.46**	0.55	0.50	148.9 mmHg
**Classification using non-imaging factors**				
NI SVM classifier	**0.74**	0.58	0.67	0.5
**NI RF classifier**	0.73	**0.74**	**0.74**	0.6
**CMB lesion count: Imaging-based methods**				
**Proposed pipeline**	**0.91**	**0.86**	**0.89**	35 CMBs
Thresholding + postproc	0.67	0.72	0.69	56 CMBs
**Classification using non-imaging factors + CMB lesion count**				
NI+I SVM classifier	0.82	0.89	0.85	0.5
**NI+I RF classifier**	**0.95**	**0.95**	**0.95**	0.6

3 Performance values at a subject-level TPR value of 95%

Table S3 shows the subject-level specificity and accuracy values determined from the ROC curves at a subject-level TPR value of 95%.

Table S3: Performance values determined from the ROC curves at a subject-level TPR value of 95% for leave-one-out validation within datasets (section 3.4.1), generalisability across datasets (section 3.4.2) and comparison of methods using imaging (I) and non-imaging (NI) factors on the UKBB datasets (3.4.3), along with the threshold values applied to get a subject-level TPR value of 95%.

**Methods**	**Sub. Spec**	**Sub. Acc**	**Threshold values**
**Leave-one-out validation within datasets (Section 3.4.1)**			
Within OXVASC	0.84	0.90	31 CMBs
Within TICH2	0.76	0.85	26 CMBs
**Generalisability experiments across datasets (Section 3.4.2)**			
OXVASC-trained model on TICH2	0.10	0.53	18 CMBs
TICH2-trained model on OXVASC	0.46	0.71	30 CMBs
OXVASC-trained model on UKBB	0.21	0.59	17 CMBs
TICH2-trained model on UKBB	0.40	0.62	26 CMBs
**Comparison of various methods on the UKBB dataset (Section 3.4.3)**			
Age	0.05	0.50	47.6 yrs
**Diastolic BP**	0.18	0.57	91.2 mmHg
Systolic BP	0.16	0.55	151.1 mmHg
NI SVM classifier	0.23	0.59	0.25
**NI RF classifier**	0.65	0.80	0.45
**Proposed pipeline**	0.82	0.89	33 CMBs
Thresholding + postproc	0.12	0.54	42 CMBs
NI+I SVM classifier	0.70	0.83	0.47
**NI+I RF classifier**	0.95	0.95	0.6

References

[1] Steven M Greenberg, Meike W Vernooij, Charlotte Cordonnier, Anand Viswanathan, Rustam Al-Shahi Salman, Steven Warach, Lenore J Launer, Mark A Van Buchem, Monique MB Breteler, Microbleed Study Group, et al. Cerebral microbleeds: a guide to detection and interpretation. *The Lancet Neurology*, 8(2):165–174, 2009.

[2] Andreas Charidimou and David J Werring. Cerebral microbleeds: detection, mechanisms and clinical challenges. *Future Neurology*, 6(5):587–611, 2011.

[3] R L Mittl, R I Grossman, J F Hiehle, R W Hurst, D R Kauder, T A Gennarelli, and G W Alburger. Prevalence of MR evidence of diffuse axonal injury in patients with

mild head injury and normal head CT findings. *American Journal of Neuroradiology*, 15(8):1583–1589, 1994.

[4] Charlotte Cordonnier, Joanna Wardlaw, and Rustam Al-Shahi Salman. Spontaneous brain microbleeds: systematic review, subgroup analyses and standards for study design and reporting. *Brain*, 130(8):1988–2003, 02 2007.
